# First person – Kyle Wegner

**DOI:** 10.1242/bio.042333

**Published:** 2019-03-15

## Abstract

First Person is a series of interviews with the first authors of a selection of papers published in Biology Open, helping early-career researchers promote themselves alongside their papers. Kyle Wegner is first author on ‘Edar is a downstream target of beta-catenin and drives collagen accumulation in the mouse prostate’, published in BIO. Kyle is a PhD candidate in the lab of Chad M. Vezina at the University of Wisconsin-Madison, investigating principles of toxicology and urology to evaluate mechanisms of urinary dysfunction in aging men.


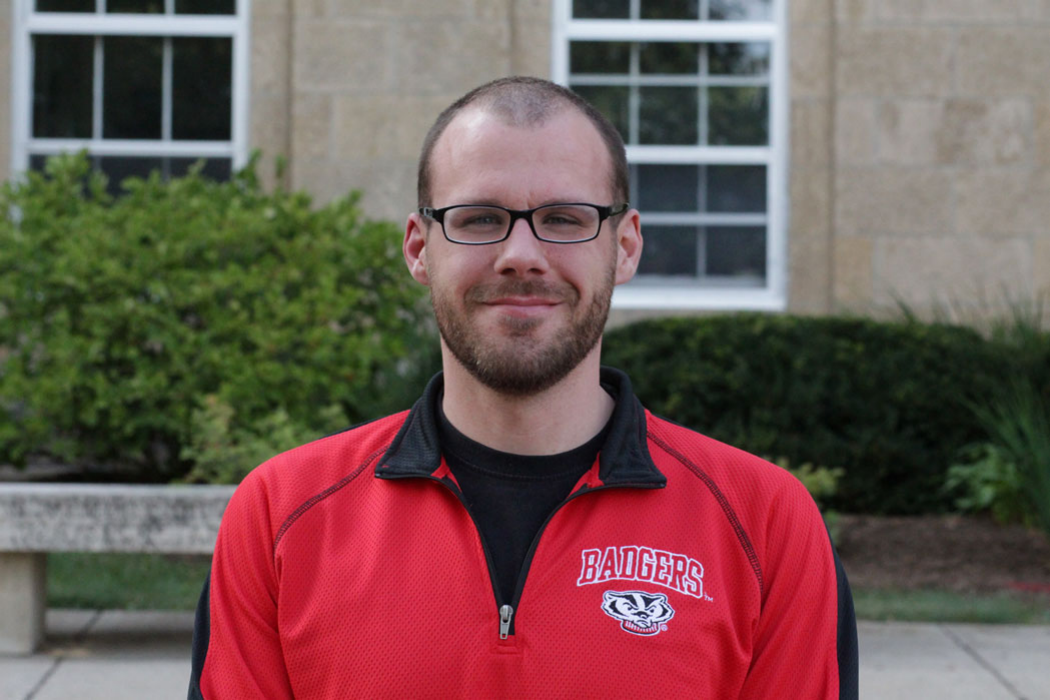


**Kyle Wegner**

**What is your scientific background and the general focus of your lab?**

My graduate research activities are focused on toxicology, a discipline merging my interests and abilities in biology and chemistry. I am a PhD candidate in the lab of Dr Chad Vezina, where we examine the etiology of urinary dysfunction in aging men. The fields of toxicology and urology have developed in parallel but have not intersected. This is unfortunate because modern toxicology principles may hold the key to identifying previously unrecognized contributors to urinary tract disease. For example, many urology researchers are surprised by the notion that a fetal chemical exposure could drive urinary dysfunction in an elderly man. My dissertation research unites principles of toxicology and urology to evaluate the mechanism of urinary dysfunction in aging men. Lower urinary tract symptoms (LUTS) associated with urinary dysfunction are among the most widespread disorders affecting aging men and include increased frequency, urgency, and difficulty of urination. Prostate enlargement has been the focus of LUTS research and treatment for decades, yet it is still unknown why time of symptom onset varies widely and why some men experience severe symptoms independent of prostate size changes. My research addresses this by testing a potential mechanism whereby early life exposure to environmental contaminants predisposes mice to urinary dysfunction. A growing body of evidence supports the hypothesis that the effects on urinary function following perinatal exposure to these chemicals are mediated by prostate fibrosis.

**How would you explain the main findings of your paper to non-scientific family and friends?**

Understanding how the prostate gland is formed and patterned is essential for understanding prostate development and long-term health. There are certain biological signals that control development of skin and hair. We asked whether these signals are also used in prostate development. Our results indicate that some signals are used during prostate development, but the process is quite different than for skin and hair. Interestingly we also found a new role for these signals in maintaining prostate health during adulthood. These signals appear to control deposition of collagen in the prostate. This is important because excessive accumulation of collagen can cause obstruction of the urethra and aging-related urinary dysfunction. Understanding how this process occurs is the first step towards designing better therapies.

**What are the potential implications of these results for your field of research?**

These results are very exciting given the recent interest in prostate fibrosis as a driver of urinary dysfunction, for which a mechanism remains unknown. Pro-collagen signaling and subsequent collagen production may be responsible for bothersome urinary symptoms in aging men and may hold the key to new therapies.

“The longstanding hypothesis that prostate enlargement is the only cause of LUTS is a real problem in clinical practice because it has stagnated therapeutic development.”

**What, in your opinion, are some of the greatest achievements in your field and how has this influenced your research?**

Most older men develop lower urinary tract symptoms (LUTS), but onset age and severity varies, suggesting a potential environmental influence. Surprisingly, environment has been largely ignored as a potential cause of these symptoms. The longstanding hypothesis that prostate enlargement is the only cause of LUTS is a real problem in clinical practice because it has stagnated therapeutic development. Recent studies have shown that changes in prostate collagen characteristics are so common in men and animals with urinary dysfunction that it has been proposed that prostate collagens/fibrosis drive LUTS. Given that prostate fibrosis is a new endpoint for urologic research, the tools required to explore this phenomenon did not exist. I used this as inspiration to develop of a new method for imaging and quantifying collagen in tissue sections. My method changes the way we look at collagen and now instead of just quantifying gross content, we can identify individual fibers, assess their characteristics and overlay them with molecular features of interest. This type of innovation is important to lay the foundation so this new field of prostate fibrosis. As such, I have also outlined a novel, immunohistochemical methodology which uses a validated polytomous key to identify immunolabeled cell types in intact sections of adult mouse prostate and prostatic urethra and software which enables objective quantification of a physiologic assay for mouse urinary function. With these tools we can now better identify urinary dysfunction in mice, objectively quantify prostate fibrosis and more accurately identify the cells producing collagen.

**Figure:**
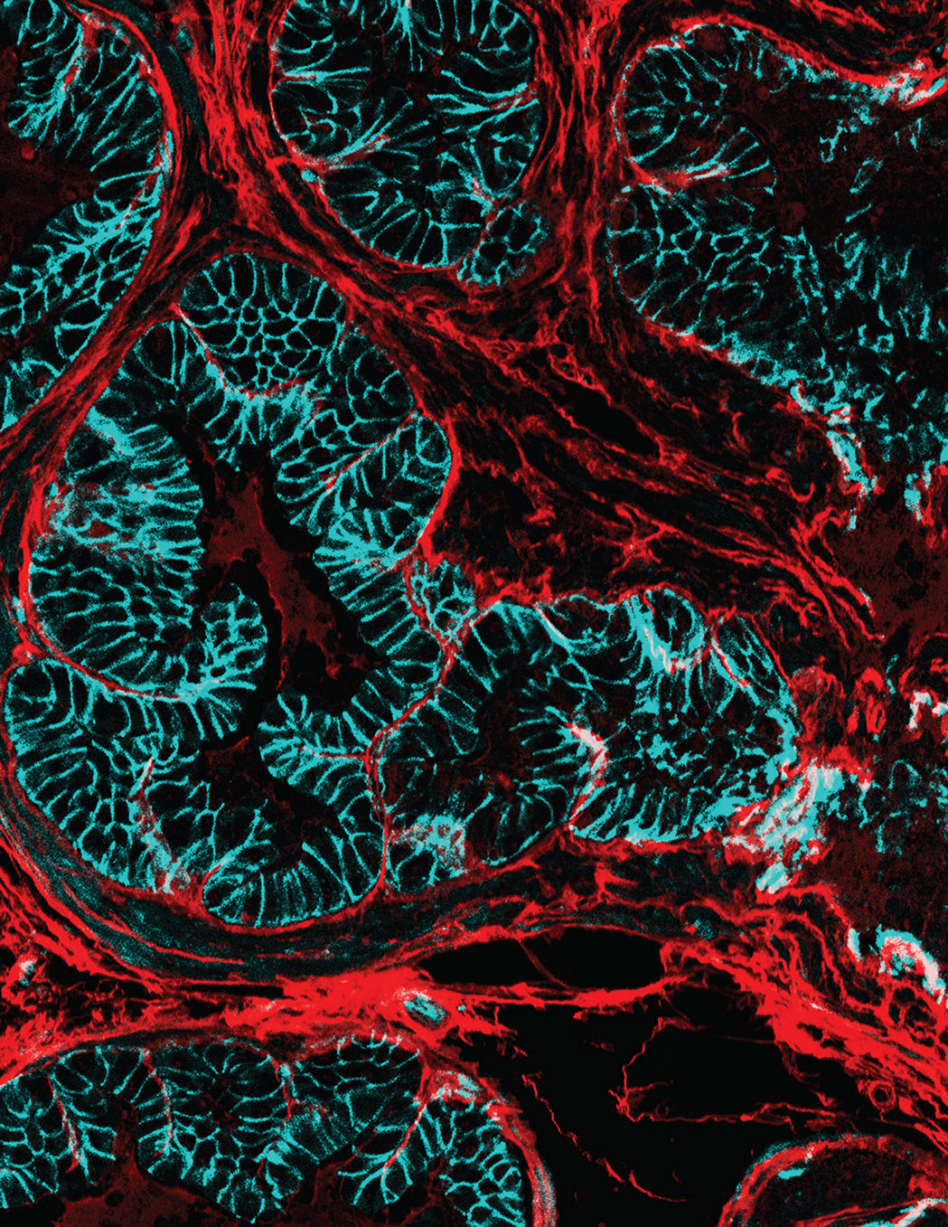
**I use fluorescent imaging and Picrosirius Red to detect collagen fibers in tissue sections.** Here, collagen fibers (red) are shown to be prevalent between ducts of prostate epithelial cells (cyan). These techniques enable me to quantify individual collagen fiber metrics (length, diameter, orientation) and how these fibers interact with surrounding cellular environment.

**What changes do you think could improve the professional lives of early-career scientists?**

My scientific career was born under the Wisconsin Idea, and as such I have made it my mission to always find ways to demonstrate the real-world value of my scientific endeavors. However, I have found that people cannot value what they do not understand or appreciate as applicable to their lives. It is the responsibility of early stage scientists to hone our skills beyond benchwork and learn how we can best share our enthusiasm for science with the public. Throughout my scientific career, I have seen how effective communication empowers an audience and frames science advocacy as a conversation rather than a lecture. Scientists who can articulate the core values of their research to those with limited scientific backgrounds invite audiences to form their own opinions, ask their own questions and discover their own inner scientists. I believe that these scientists are the most effective advocates we have and are a source of inspiration for me.

**What's next for you?**

With my graduation occurring in fall 2019, I am actively looking for my next position and I am eager to connect with other researchers and industry professionals. My goal is to serve as an independent toxicological researcher in the private sector. Combining deep knowledge of science with the exciting challenges of an industry environment greatly appeals to me. Feel free to contact me if you would like to connect!
